# Inflammasome activation aggravates choroidal neovascularization

**DOI:** 10.1007/s10456-024-09949-1

**Published:** 2024-09-24

**Authors:** Ryan D. Makin, Ivana Apicella, Roshni Dholkawala, Shinichi Fukuda, Shuichiro Hirahara, Yoshio Hirano, Younghee Kim, Ayami Nagasaka, Yosuke Nagasaka, Siddharth Narendran, Felipe Pereira, Akhil Varshney, Shao-bin Wang, Jayakrishna Ambati, Bradley D. Gelfand

**Affiliations:** 1 Center for Advanced Vision Science, University of Virginia School of Medicine, Charlottesville, VA 22903, USA; 2 Department of Ophthalmology, University of Virginia School of Medicine, Charlottesville, VA 22903, USA; 3 Molecular and Cellular Basis of Disease Graduate Program, University of Virginia School of Medicine, Charlottesville, VA 22903, USA; 4 Department of Ophthalmology, University of Tsukuba, Tsukuba 305-8575, Ibaraki, Japan; 5 Department of Ophthalmology and Visual Science, Nagoya City University Graduate School of Medical Sciences, Nagoya, Japan; 6 Aravind Eye Care System, Madurai, India; 7 Departamento de Oftalmologia e Ciências Visuais, Escola Paulista de Medicina, Universidade Federal de São Paulo, São Paulo, Brazil; 8 Department of Pathology, University of Virginia School of Medicine, Charlottesville, VA 22903, USA; 9 Department of Microbiology, Immunology, and Cancer Biology, University of Virginia School of Medicine, Charlottesville, VA 22903, USA; 10 Department of Biomedical Engineering, University of Virginia School of Engineering, Charlottesville, VA 22903, USA

**Keywords:** Choroidal neovascularization, Inflammasome, Macrophage, Age-related macular degeneration, Myd88, Interleukin-1beta

## Abstract

Inflammasome activation is implicated in diseases of aberrant angiogenesis such as age-related macular degeneration (AMD), though its precise role in choroidal neovascularization (CNV), a characteristic pathology of advanced AMD, is ill-defined. Reports on inhibition of inflammasome constituents on CNV are variable and the precise role of inflammasome in mediating pathological angiogenesis is unclear. Historically, subretinal injection of inflammasome agonists alone has been used to investigate retinal pigmented epithelium (RPE) degeneration, while the laser photocoagulation model has been used to study pathological angiogenesis in a model of CNV. Here, we report that the simultaneous introduction of any of several disease-relevant inflammasome agonists (*Alu* or B2 RNA, *Alu* cDNA, or oligomerized amyloid β (1–40)) exacerbates laser-induced CNV. These activities were diminished or abrogated by genetic or pharmacological targeting of inflammasome signaling constituents including P2rx7, Nlrp3, caspase-1, caspase-11, and Myd88, as well as in myeloid-specific caspase-1 knockout mice. *Alu* RNA treatment induced inflammasome activation in macrophages within the CNV lesion, and increased accumulation of macrophages in an inflammasome-dependent manner. Finally, IL-1β neutralization prevented inflammasome agonist-induced chemotaxis, macrophage trafficking, and angiogenesis. Collectively, these observations support a model wherein inflammasome stimulation promotes and exacerbates CNV and may be a therapeutic target for diseases of angiogenesis such as neovascular AMD.

## Introduction

Inflammasome activation is implicated in the pathogenesis of a variety of complex diseases, including retinal diseases such as age-related macular degeneration (AMD) and diabetic retinopathy. In the context of AMD, evidence supports that inflammasome activation promotes atrophic retinal degeneration in cell and animal models and geographic atrophy [1–4], the advanced form of dry AMD.

Conversely, neovascular AMD is characterized by aberrant growth of blood vessels into the outer retina, which is normally avascular. These pathological neovessels typically emerge from the underlying choroid or inner retina. Mice lacking inflammasome constituents are protected against spontaneous choroidal neovascularization (CNV) in mouse models driven by an excess of VEGFA or DICER1 deficiency [5, 6]. Conversely, the role of inflammasome in laser photocoagulation induced CNV, the benchmark model of choroidal angiogenesis which is driven by a thermal injury burn to the pigmented epithelium that results in an acute angiogenic and wound healing response, lacks consensus. Initial reports suggested that *Nlrp3*^−/−^ mice exhibit elevated laser-induced CNV [7], though this finding was challenged by a multinational consortium which found that genetic or pharmacologic inhibition of core inflammasome constituents or effectors does not increase experimental CNV [8]. Still others have reported that pharmacologic inhibition of caspase-1 suppresses CNV [5].

It is conceivable that laser photocoagulation, an artificial injury stimulus, does not consistently stimulate or rely on inflammasome to induce angiogenesis, which may contribute to the heterogeneous findings of inflammasome in genetic/spontaneous and injury/acute models of CNV. Therefore, we sought to investigate the role of inflammasome activation in CNV by introducing a new model in which subretinal administration of disease-relevant inflammasome stimulators is performed with laser induced CNV. Using this novel model, we report that while inflammasome constituents are dispensable for the laser CNV response, the addition of inflammasome stimulators exacerbates pathologic choroidal angiogenesis, and that in the presence of inflammasome agonists, genetic or pharmacologic intervention of inflammasome signaling significantly improves CNV outcomes in mice.

## Materials and methods

### Mice

All experiments involving animals were approved by the University of Virginia Animal Care and Use Committee and in accordance with the Association for Research in Vision and Ophthalmology Statement for the Use of Animals in Ophthalmic and Visual Research.

Mice were maintained on a constant 12:12-h light–dark cycle. Water and food were provided ad libitum. Mice were euthanized with CO2 gas under constant gas flow. C57BL/6J wild-type, *P2rx7*^*−/−*^, *Casp1^−/−^*/*11*^−/−^, *LysM-Cre*, and *Aim2*^−/−^ mice were obtained from The Jackson Laboratory. *Casp1*^−/−^/*11*^−/−^;*Casp11_Tg_* mice, described elsewhere [9], were a generous gift from V. M. Dixit (Genentech, South San Francisco, California). *Myd88*^−/−^ mice were generously provided by S. Akira via T. Hawn and D. T. Golenbock. *Casp1^f/f^* mice were a generous gift from Dr. Richard Flavell (Yale University). *Nlrp3*^−/−^ mice have been previously described [10]. For all procedures, anesthesia was achieved by intraperitoneal injection of 100 mg/kg ketamine hydrochloride (Ft. Dodge Animal Health, Overland Park, Kansas, US) and 10 mg/kg xylazine (Phoenix Scientific, San Marcos, California, US), and pupils were dilated with topical 1% tropicamide and 2.5% phenylephrine (Alcon Laboratories, Elkridge, Maryland, US).

### Laser-induced CNV and subretinal injection

**Fig. 1:**
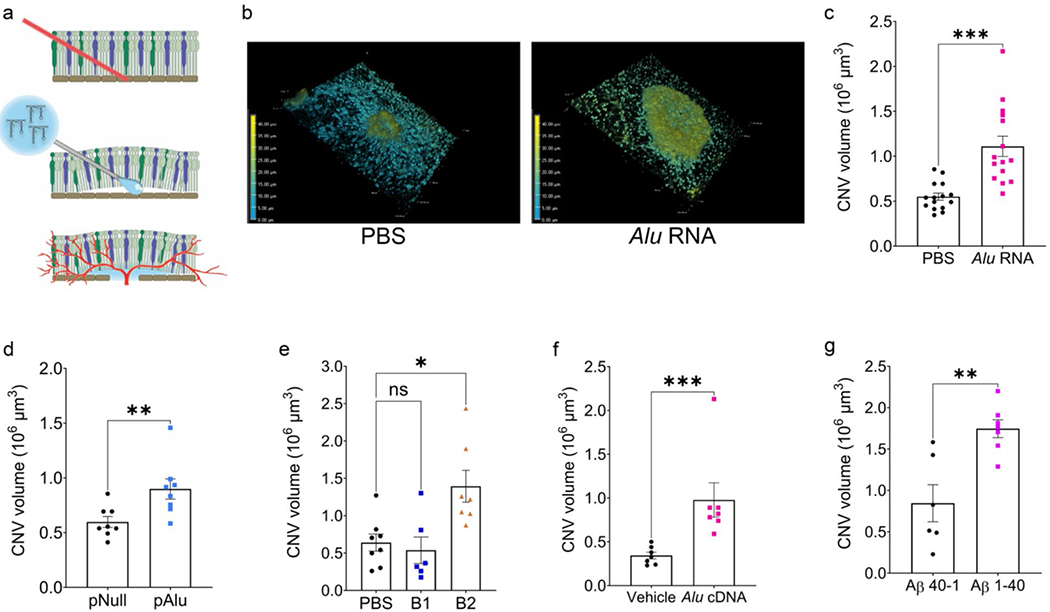
Inflammasome agonism immediately following laser injury increases CNV volume. (**a**) Schematic demonstrating the combined laser CNV and subretinal injection (SRI) model. First, a laser burn is applied to rupture Bruch’s membrane. Subretinal injection is performed immediately following laser injury at the same site, and neovascularization begins to form around day 3. (**b**) Representative depth-coded 3D projections of laser CNV with SRI of PBS (left) or *Alu* RNA (right). Dimensions: 633.25 μm x 633.25 μm x 46 μm (**c**) CNV volumes quantified 7 days after combined laser injury and SRI *Alu* RNA (*P* = 0.0001, Mann-Whitney test. *N* = 15 per group). (**d**) CNV volumes quantified 7 days after combined laser injury and in vivo transfection of plasmid-encoded *Alu* via SRI (*P* < 0.01, Mann-Whitney test. *N* = 8 per group). (**e**) CNV volumes quantified 7 days after combined laser injury and SRI of PBS, B1 (*P* > 0.99 vs. PBS), or B2 RNA (*P* = 0.03 vs. PBS, Kruskal-Wallis test. *N* = 6 (B1), *N* = 7 (B2)). (**f**) CNV volumes quantified 7 days after combined laser injury and SRI *Alu* cDNA (*P* = 0.01, Mann-Whitney test. *N* = 1 (vehicle), *N* = 7 (*Alu* cDNA)). (**g**) CNV volumes quantified 7 days after combined laser injury and SRI of Aβ (*P* < 0.01, Mann-Whitney test. *N* = 6 (Aβ_40–1_), *N* = 7 (Aβ_1–40_))

A schematic of the laser-induced CNV and injection procedure is depicted in Fig. 1a. Experimental CNV was induced by performing a single laser photocoagulation burn with an OcuLight GL laser system (532 nm, 180 mW, 100 ms, 75 μm; Iridex Corporation, Mountain View, CA, USA) bilaterally in 6- to 8-week-old mice. Immediately following laser CNV, subretinal injection of experimental compounds was performed as described previously [11]. Briefly, a 5 μl microsyringe was filled with 1–2 μl of reagent and attached to a custom 37G needle. The needle was then introduced at a 60° angle until it touched the retina and a retinotomy was induced to access the subretinal space. The reagent was then slowly dispensed into the subretinal space such that the resulting detachment encompassed the area previously treated with the laser. The following reagents were used for subretinal injection: PBS (VWR, Radnor, PA, US; 97063-660); in vitro transcribed *Alu*, B1, and B2 RNAs as previously described [12]; plasmid-encoded *Alu* RNA and empty plasmid control with NeuroPORTER Transfection Reagent (AMSBIO, Cambridge, MA, US; AMS. T400150) as previously described [13]; amyloid-β as previously described [14]. For intravitreous injections the following reagents were administered immediately following laser thermal injury and subretinal injections: azidothymidine (AZT), 0.5 nmol (Selleck Chemicals, Houston, TX, US; S2579); 2-ethyl-AZT, 0.5 nmol (previously described in [14]); Z-WEHD-FMK (R&D, Minneapolis, MN, US; FMK002) or control peptide Z-FA-FMK (R&D; FMKC01); IL-1β neutralizing antibody, 500 ng (R&D, MAB4012) or isotype IgG, 500 ng (Thermo, 14-4888-81); MyD88 inhibitory peptide or control peptide (Novus, Centennial, CO, US; NBP2-29328-1 mg).

### RPE/choroid flatmount preparation and CNV volume quantification

RPE flatmounts were obtained as previously described [11]. At the indicated timepoint, eyes were enucleated and fixed in 4% paraformaldehyde/PBS for 1 h at room temperature. After removal of the cornea, lens, and neurosensory retina, flatmounts were dehydrated and rehydrated through a methanol series, washed in 1x PBS, and incubated in blocking buffer (1% BSA in PBST) for 1 h at 4 °C. Flatmounts were then incubated with 0.7% FITC-isolectin B4 overnight at 4 °C. Flatmounts were then washed in 1x PBS-T and mounted on glass slides with Vectashield antifade mounting medium (Vector Biolabs, Newark, CA, US; H-1000-10). CNV volumes were quantified as previously described [15].

### F4/80 immunofluorescence and quantification

For F4/80 immunofluorescence on flatmounts, eyes were processed as described above. Eyes were then incubated in rat anti-mouse F4/80:RPE (Bio-Rad, Hercules, CA, US; MCA497PE, clone Cl: A3-1) overnight at 4 °C. Washing and imaging was also performed as described above. Macrophage numbers were manually quantified by a masked grader using the FIJI plugin Cell Counter (https://imagej.net/ij/plugins/cell-counter.html) who was blinded to the experimental conditions.

### Immunofluorescence

Fresh, unfixed eyes were embedded in optimal cutting temperature medium (Tissue-Tek OCT Compound, VWR; 25608-930) and frozen in liquid nitrogen-cooled isopentane. Immunofluorescent analysis was performed by blocking 4% PFA-fixed sections with donkey block (2% normal donkey serum; 1% BSA; 0.1% Triton X-100; 0.05% Tween-20; 0.05% NaN_3_ in PBS) for 1 h at 37 °C followed by overnight incubation at 4 °C with the following antibodies: PE antimouse/human CD11b 1:50, clone M1/70 (BioLegend, San Diego, CA, US; 101207); anti-cleaved caspase-1 (Asp296) 1:100, clone E2G2I (Cell Signaling Technology, Danvers, MA, US; 89332). Anti-cleaved caspase-1 antibodies were detected with Alexa Fluor 647-conjugated donkey anti-rabbit secondary antibody (Thermo, A-31573) at a concentration of 1:1000 in donkey block. Equivalent amount of rabbit IgG was used for isotype control.

### Fluorescent *in situ* hybridization

Fresh-frozen cryosections prepared as above were probed with the RNAscope Multiplex Fluorescent Reagent Kit v2 (ACDBio, Newark, CA) according to manufacturer’s instructions using the following probes: Mm-Il1b (# 316891); Mm-Adgre1-C2 (# 460651-C2); Mm-P2ry12-C3 (# 317601-C3). Probes were detected with the following fluorophores diluted 1:1000 in RNAscope TSA buffer: Opal 520 (Akoya Biosciences, Marlborough, MA, # FP1487001KT); Opal 570 (Akoya, # FP1488001KT); Opal 650 (Akoya, # FP1496001KT). Imaging was performed on a Nikon A1R confocal microscope.

### Transwell migration assays

The chemotactic ability of WT BMDM was assessed using 8.0 μm permeable polycarbonate inserts (Celltreat, Pepperell, MA; 230633). Four hours before addition of chemotactic agent, 600 μL 2% BMDM media was added to the bottom well of a 24-well plate and 60,000 cells in 100 μL 2% BMDM media were seeded into the insert. After four hours, media from the bottom well was aspirated and replaced with the chemoattractant under study and incubated at 37 °C/5% CO_2_ for twelve hours. The following chemoattractants were used: recombinant mouse VEGF_164_ protein, 50 ng/mL (R&D; 493-MV-005); conditioned media from *Alu* RNA-transfected WT and *Casp1*^−/−^ BMDM, diluted to 10% in RPMI (ThermoFisher; 12440061). Inserts were then rinsed three times in 1x PBS, unmigrated cells on the apical side of the transwell scraped with a cotton-tipped applicator, and fixed in 4% PFA/PBS for one hour. After rinsing three times in 1x PBS, membranes were excised with a scalpel and mounted on glass slides in ProLong Gold Antifade with DAPI (ThermoFisher, P36935). For each membrane, five fields of view at 20X were imaged with a Nikon Eclipse Ti2 inverted widefield fluorescence microscope and quantified in FIJI (http://fiji.sc/).

### Statistics

Using empirical data on the variability of PBS-injected laser CNV lesions (Fig. 1c), power analysis determined that a minimum of *N* = 5 eyes are needed to detect a 50% change in CNV volume with 80% power. Experiments were designed to exceed this to account for technical complications. Statistical analyses were performed using GraphPad Prism (GraphPad Software, Version 9.1.2, San Diego, CA, US). Unless otherwise stated, results are shown as mean ± standard error of mean. *P* values of less than 0.05 were deemed statistically significant by either two-tailed Mann Whitney U test, two-tailed Kruskal-Wallis test, or one- or two-way ANOVA with multiple comparisons corrections, as stated in the figure legends.

## Results

### Inflammasome agonists exacerbate experimental CNV

To test the hypothesis that inflammasome activators promote excess choroidal angiogenesis, we adapted the laser photocoagulation model by applying a single laser burn to wild-type C57BL/6J mice eyes followed immediately by subretinal injection of PBS or *Alu* RNA, which is an AMD-related inflammasome agonist transcribed from short interspersed nuclear element (SINE) retrotransposons [2, 13] (Fig. 1a). Seven days later, *Alu* RNA-treated eyes exhibited a dramatic increase in the volume of the CNV lesion compared to saline-treated eyes (Fig. 1b, c). Similarly, subretinal administration of a plasmid expressing *Alu* RNA (pAlu) with transfection reagent resulted in an increased CNV response compared to the transfection of an empty plasmid (pNull) (Fig. 1d, S1).

Administration of murine SINE B2 RNA, which like *Alu* RNA is an inflammasome agonist [2, 13], also exacerbated CNV (Fig. 1e, S1). Interestingly, mouse SINE B1 RNA, which is a poorer inflammasome agonist [12], did not significantly affect CNV (Fig. 1e, S1).

*Alu* RNA can be reverse transcribed by the LINE-1 reverse transcriptase into a complementary DNA (*Alu* cDNA) which stimulates inflammasome activation, promotes RPE death, and is enriched in the retina of human AMD eyes [16, 17]. Subretinal delivery of synthetic *Alu* cDNA also increased the volume of CNV lesions (Fig. 1f, S1). To assess whether this angiostimulatory property was unique to SINE-derived oligonucleotides, we tested amyloid-β, another inflammasome agonist that accumulates in human AMD [18–20], contributes to spontaneous CNV in a mouse model [21], and promotes inflammasome-dependent RPE death [14, 22]. Subretinal delivery of oligomerized amyloid-β_1–40_, but not a control peptide (amyloid-β_40–1_), likewise aggravated CNV (Fig. 1g, S1). These findings indicate that multiple inflammasome agonists of different compositions can amplify the choroidal angiogenic response in the laser injury model.

### The angiostimulatory activity of *Alu* RNA depends on inflammasome

As inflammasome agonists may have pleiotropic effects aside from inflammasome activation, we next sought to determine whether this angiostimulatory effect was indeed dependent on inflammasome activity. To test this, we administered *Alu* RNA immediately following laser injury in mice lacking constituents of the inflammasome pathway, or in wild-type mice treated with pharmacologic inflammasome inhibitors.

**Fig. 2:**
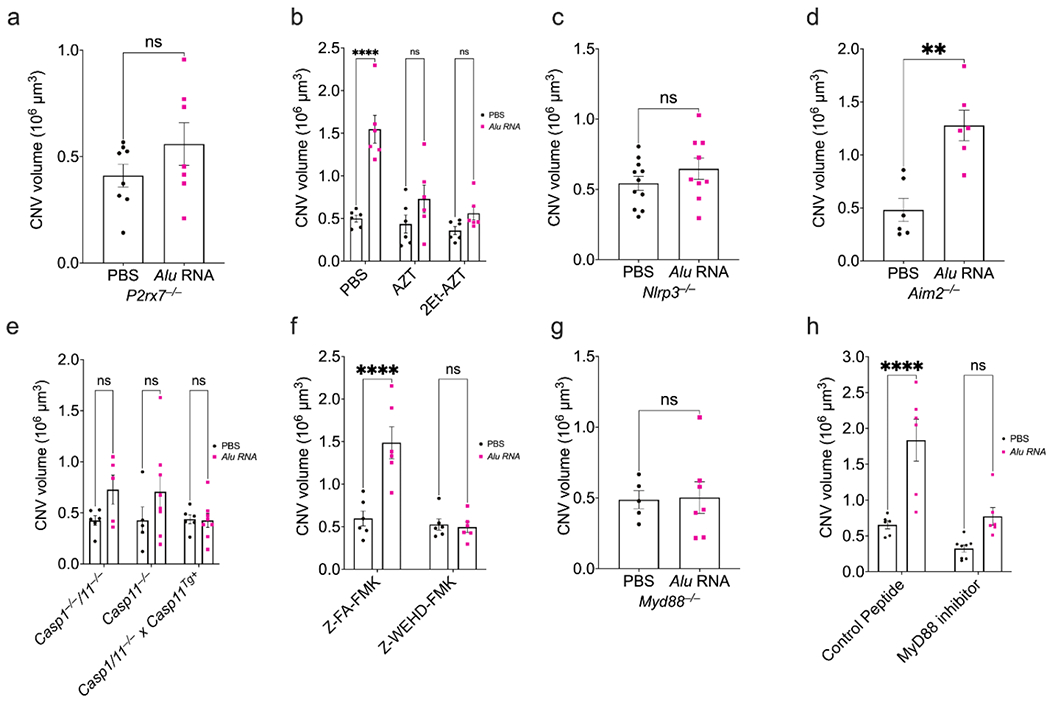
Intact NLRP3 inflammasome components are required for inflammasome agonism-dependent CNV exacerbation. (**a**) CNV volumes quantified 7 days after combined laser injury and SRI of *Alu* RNA in *P2rx7*^−/−^ mice (*P* > 0.99, Mann-Whitney test. *N* = 7–8). (**b**) CNV volumes quantified 7 days after combined laser injury, SRI of *Alu* RNA, and intravitreous pretreatment with PBS (*P* < 0.01), AZT (*P* = 0.20), or K8 (*P* = 0.50) (two-way ANOVA. *N* = 6 per group). (**c**) Quantification of CNV volume 7 days post *Alu* RNA SRI in *Nlrp3*^−/−^ mice (*P* = 0.412, Mann-Whitney test. *N* = 11 (PBS). *N* = 9 (*Alu* RNA)). (**d**) CNV volumes quantification 7 days after laser injury and SRI of *Alu* RNA in *Aim2*^−/−^ mice (*P* < 0.01, Mann-Whitney test. *N* = 6 per group). (**e**) CNV volumes quantified after combined laser injury and SRI of *Alu* RNA in *Casp1/11*^−/−^ (*P* = 0.25), *Casp11*^−/−^ (*P* = 0.25). and *Casp1/11*^−/−^ x *Casp11^Tg^*^+^ (*P* > 0.99) (two-way ANOVA, *N* ≥ 5 per group). (**f**) CNV volume quantification 7 days after combined laser injury, intravitreous administration of either control peptide Z-FA-FMK (*P* < 0.01) or caspase-1 inhibitor Z-WEHD-FMK (*P* = 0.98), and *Alu* RNA SRI (two-way ANOVA, *N* = 6 per group). (**g**) CNV volumes quantified after 7 days post-laser injury and *Alu* RNA SRI in *Myd88*^−/−^ mice (*P* = 0.79, Mann-Whitney U test, *N* ≥ 5 per group). (**h**) CNV volumes quantified 7 days after combined laser injury, intravitreous administration of a peptide MyD88 inhibitor (*P* = 0.08) or control peptide (*P* < 0.01). and *Alu* RNA SRI (*P* = 0.08. two-way ANOVA, *N* ≥ 6 per group)

The ATP receptor P2X7 is an upstream driver of inflammasome activation and is required for *Alu* RNA-induced RPE degeneration [23]. In *P2rx7*^−/−^ mice, *Alu* RNA did not exacerbate laser-induced CNV (Fig. 2a). Nucleoside reverse transcriptase inhibitors (NRTIs) prevent P2X7-dependent inflammasome activation and RPE degeneration [14, 15, 24, 25]. Administering the NRTI zidovudine (AZT) into the vitreous of wild-type mice immediately following thermal laser burn and subretinal *Alu* RNA injection also abrogated the angiostimulatory response (Fig. 2b). In addition to targeting P2X7, NRTIs also inhibit reverse transcriptases. A 2-ethoxylated-modified derivative of AZT that does not inhibit reverse transcriptase but retains anti-inflammatory activities [25] also blunted *Alu* RNA-induced CNV (Fig. 2b), further supporting that the angiostimulatory activity of *Alu* RNA depends on P2X7.

Downstream of P2X7 activation, *Alu* RNA stimulates inflammasome assembly consisting of NLRP3, ASC, and the protease caspase-1 [2]. As in *P2rx7*^−/−^ mice, CNV lesions in mice lacking NLRP3 (*Nlrp3*^−/−^) were also unaffected by *Alu* RNA (Fig. 2c). Conversely, mice lacking AIM2 (*Aim2*^−/−^) an alternative inflammasome receptor that does not mediate *Alu* RNA-induced RPE death (Fig S2), were susceptible to *Alu* RNA-induced exacerbation of CNV (Fig. 2d).

We next investigated the contribution of caspase-1, the inflammasome effector protease. Mice lacking both caspase-1 and the non-canonical inflammasome effector caspase-11 (*Casp1*^−/−^;*Casp11*^−/−^) are resistant to *Alu* RNA-induced RPE degeneration [2]. Similarly, *Casp1*^−/−^/*Casp11*^−/−^ mice were resistant to the angiostimulatory activity of *Alu* RNA in CNV (Fig. 2e). In double knockout mice in which caspase-11 expression is rescued by a transgene *Casp1*^−/−^; *Casp11*^−/−;Tg+^), *Alu* RNA treatment did not affect CNV (Fig. 2e), supporting that caspase-1 is essential for *Alu* RNA stimulated CNV. Furthermore, administration of Z-WEHD-FMK, a cell-permeable irreversible inhibitor of caspase-1, into the vitreous humor of wild-type mice also abrogated the angiostimulatory effect of *Alu* RNA on laser CNV (Fig. 2f). RPE degeneration by *Alu* RNA also depends on the activity of caspase-11 [26]. Mice lacking just caspase-11 (*Casp11*^−/−^) were partially protected against *Alu* RNA-induced CNV exacerbation (Fig. 2f), indicative of some contribution of non-canonical inflammasome activation to this process.

Inflammasome activation results in maturation of the effector cytokines IL-1β and IL-18, whose signal transduction requires the adaptor MyD88 [27]. Mice lacking MyD88 (*Myd88*^−/−^) are protected against *Alu* RNA-induced RPE degeneration [2]. In *Myd88*^−/−^ mice, administration of *Alu* RNA did not affect CNV volume (Fig. 2g). Additionally, whereas *Alu* RNA induced excess CNV in eyes receiving a cell-permeable control inhibitor via intravitreous injection, administration of a MYD88 homodimerization peptide inhibitor [28] diminished the effect of *Alu* RNA on laser CNV (Fig. 2h).

Collectively, these findings suggest that in the presence of an inflammasome agonist, inflammasome signaling amplifies pathological choroidal angiogenesis.

### Inflammasome in myeloid cells is critical for *Alu* RNA-induced CNV exacerbation

**Fig. 3:**
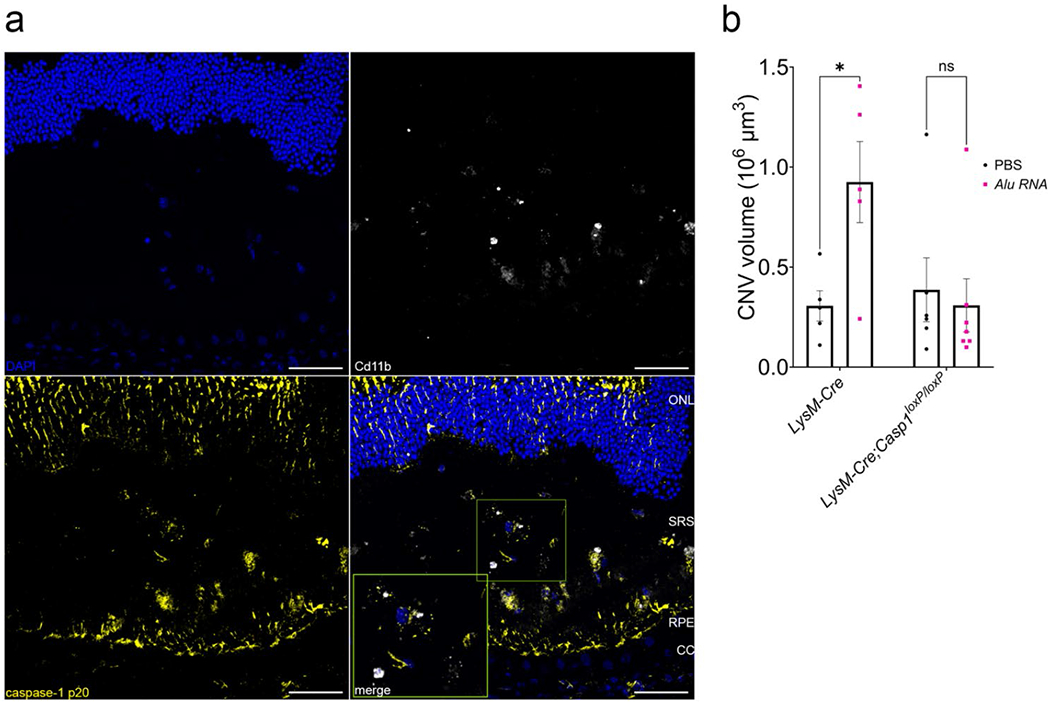
Inflammasome activation in myelomonocytic cells is crucial to laser CNV. (**a**) Representative immunofluorescence images of cross-section of mouse retinae treated with *Alu* RNA. Slides were stained with indicated antibodies. Scale bars: 50 μm. CC: choriocapillaris; RPE: retinal pigmented epithelium; SRS: subretinal space; ONL: outer nuclear layer (**b**) CNV volume quantification 7 days post laser injury and *Alu* RNA SRI in *LysM-Cre* (*P* = 0.03, *N* = 5) and *Casp1^f/f^* x *LysM-Cre* (*P* = 0.91, *N* = 6) mice (two-way ANOVA)

Laser CNV is a multicellular process involving multiple resident and recruited cells. Among these, circulating macrophages and neutrophils are recruited to CNV lesions and drive their growth [29, 30]. In *Alu* RNA-treated CNV lesions, immunofluorescent labeling revealed robust inflammasome activation via positive immunolabeling of the p20 subunit of caspase-1, which in part co-localized with CD11b^+^ macrophages (MΦ) (Fig. 3a). In addition, we observed substantial co-labeling of p20 and GFAP in Müller glia overlying the CNV lesion (Fig S3), though this p20/GFAP pattern was not as specific to the *Alu* RNA-treated CNV lesion as the p20/CD11b^+^. Therefore, we chose to assess whether inflammasome-dependent CNV expansion depends on inflammasome activation in myeloid cells, we generated myelomonocytic cell-specific caspase-1 knockout mice (*LysM-Cre*; *Casp1*^loxP/loxP^). We confirmed caspase-1 protein ablation by western blotting (Fig S4). *Alu* RNA-stimulated CNV was abrogated in *LysM-Cre*; *Casp1*^loxP/loxP^, but not in LysM-Cre-expressing control mice (*LysM-Cre*; *Casp1*^+/+^) (Fig. 3b), strongly suggesting inflammasome activation in myelomonocytic cells is responsible for *Alu*-RNA induced CNV aggravation.

### Inflammasome-dependent macrophage migration drives CNV exacerbation

**Fig. 4:**
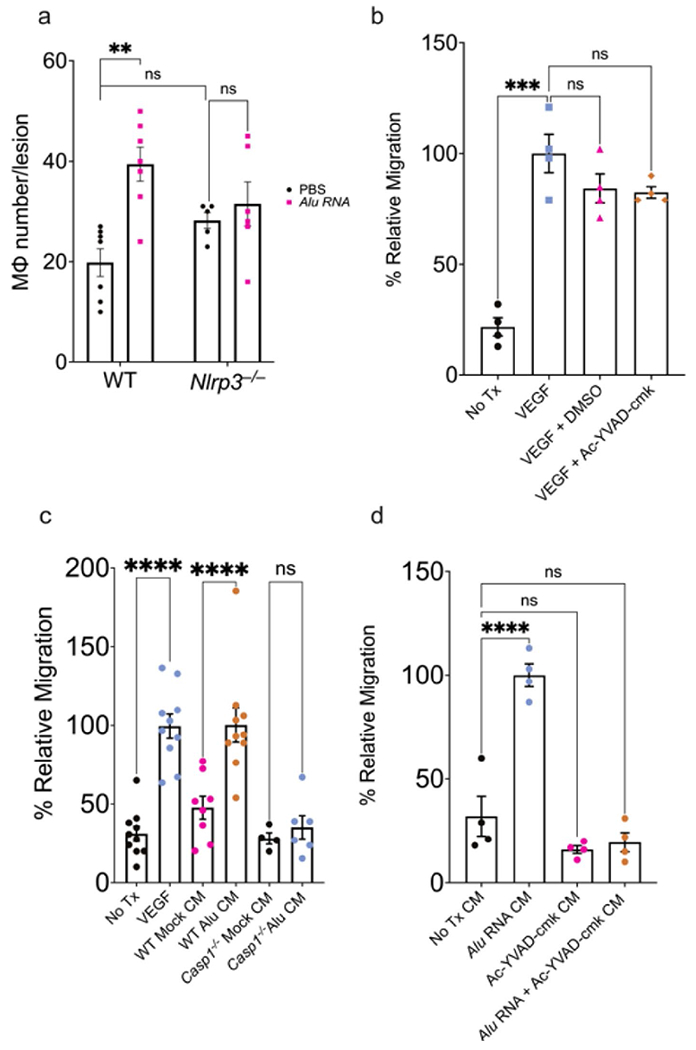
Inflammasome activation promotes chemotaxis in peripheral BMDM. (**a**) Macrophage number quantification after 3 days post laser injury and *Alu* RNA SRI in WT (*P* = 0.03, *N* = 7) and *Nlrp3*^−/−^ (*P* = 0.85, *N* = 7, two-way ANOVA). (**b**) Relative migration of WT BMDM toward the following chemoattractants: VEGF, VEGF + DMSO, VEGF + Ac-YVAD-cmk (*P* < 0.001 compared to untreated cells, *N* = 4, ordinary one-way ANOVA with Tukey’s multiple comparisons test). (**c**) Relative migration quantification of *Alu* RNA-transfected WT BMDM conditioned media (*P* < 0.01, *N* ≥ 8) and Alu RNA-transfected *Casp1*^−/−^ BMDM (*P* = 0.97, *N* ≥ 4, ordinary one-way ANOVA with Tukey’s multiple comparisons test). (**d**) Relative migration of WT BMDM with the following conditioned media as chemoattractant: Alu RNA-transfected WT BMDM (*P* < 0.01); untransfected WT BMDM pretreated with Ac-YVAD-cmk (*P* = 0.24); Ac-YVAD-cmk pretreated, Alu RNA-transfected WT BMDM (*P* = 0.43, *N* = 4, ordinary one-way ANOVA with Tukey’s multiple comparisons test)

Based on the observation that inflammasome activation in myelomonocytic cells is required for inflammasome-induced CNV aggravation, we sought to assess whether inflammasome agonists and constituents affect macrophage recruitment in CNV. We quantified Mϕ migration in vivo by measuring the number of F4/80^+^ cells in *Alu* RNA-treated CNV lesions of WT and *Nlrp3*^−/−^ mice. In the absence of an inflammasome agonist, CNV lesions from mice lacking Nlrp3 had similar F4/80^+^ immunolabeling cell count after laser injury compared to wild-type mice (Fig. 4a; representative images shown in Fig S5). Treatment with *Alu* RNA induced a greater number of CNV-associated F4/80^+^ cells in WT but not in *Nlrp3*^−/−^ mice. Taken together, these findings suggest that inflammasome activation may mediate CNV exacerbation through the recruitment of immune cells.

To assess the contribution of inflammasome in macrophage recruitment, a Boyden chamber assay was used in which WT Mϕ were allowed to migrate towards a chemoattractant agent through a permeable support. As anticipated, a VEGF gradient stimulated robust MΦ migration (Fig. 4b). Neither DMSO nor Ac-YVAD-cmk, a cell-permeable caspase-1 inhibitor, impaired VEGF-induced chemotaxis, confirming that inflammasome inhibition did not affect the VEGF-induced chemotactic response (Fig. 4b). Next, we assessed whether inflammasome activation stimulates production of chemotactic signals. Conditioned media from *Alu* RNA-transfected wild-type MΦ stimulated chemotaxis to a similar degree as VEGF. However, conditioned media from *Casp1*^−/−^; *Casp11*^−/−^ MΦ exhibited no detectable chemotactic activity following *Alu* RNA transfection (Fig. 4c). Similarly, conditioned media from WT MΦ pretreated with the caspase-1 inhibitor Ac-YVAD-cmk no longer exhibited *Alu* RNA-induced chemotactic activity (Fig. 4d). These findings indicate that inflammasome activation stimulates the production of soluble chemotactic factors.

### Inflammasome-induced macrophage migration is mediated by interleukin-1 beta (IL-1β)

**Fig. 5:**
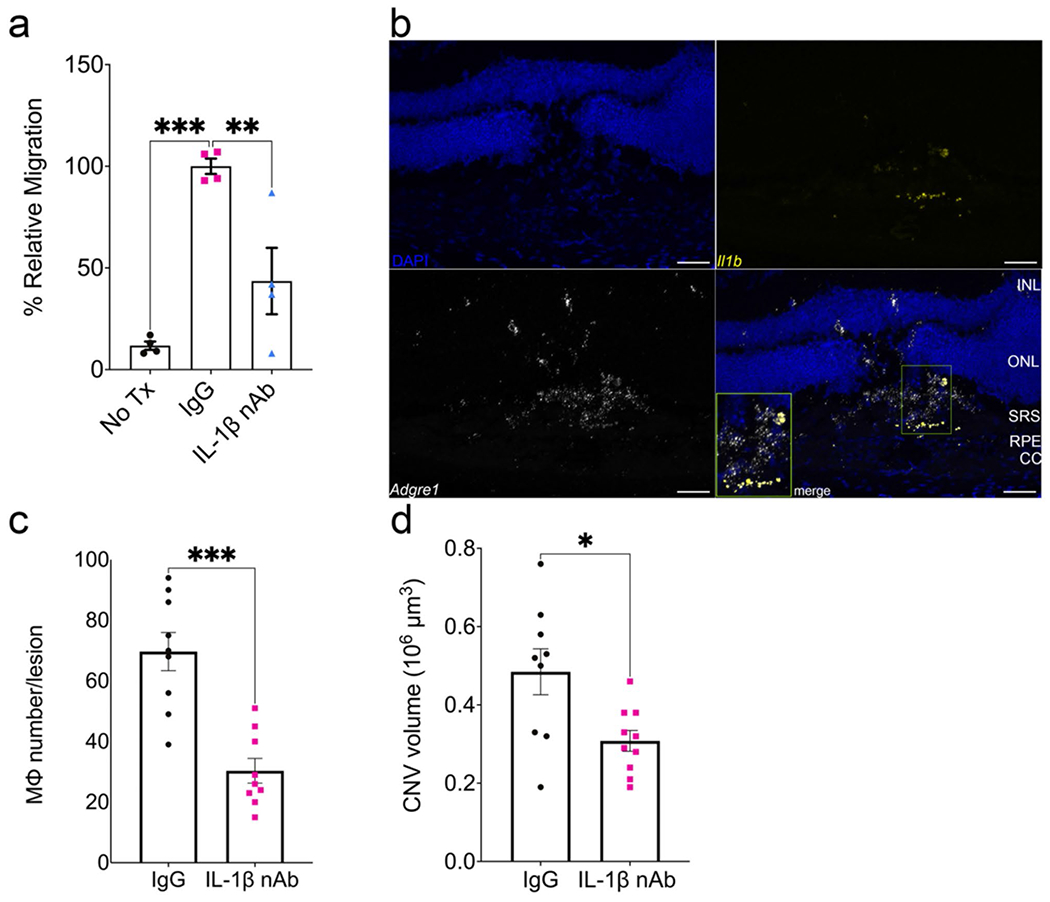
IL-1β neutralization reduces *Alu* RNA-induced chemotaxis, macrophage accumulation, and laser CNV exacerbation. (**a**) Relative migration of *Alu* RNA-transfected WT BMDM conditioned media pretreated with either IgG (*P* < 0.01) or IL-1β neutralizing antibody (*P* < 0.01, *N* = 4, ordinary one-way ANOVA with Tukey’s multiple comparisons test). (**b**) Representative images of *Alu* RNA-treated laser CNV lesions hybridized with probes against *Il1b* and *Adgre1*. Scale bar: 50 μm. CC: choriocapillaris; RPE: retinal pigmented epithelium; SRS: subretinal space; ONL: outer nuclear layer; INL: inner nuclear layer (**c**) Macrophage number and (**d**) CNV volume quantification after 3 days post laser injury and *Alu* RNA subretinal injection with either 500 ng IgG1 or IL-1β neutralizing antibody (*P* < 0.001, *N* = 8–10)

We sought to identify the inflammasome-dependent chemotactic factor responsible for macrophage migration. We focused on IL-1β as transfection of *Alu* RNA in WT MΦ induces its robust secretion [12] and it possesses chemotactic activity [31]. Conditioned media from *Alu* RNA-transfected WT BMDM incubated with an IL-1β neutralizing antibody (nAb) significantly inhibited WT BMDM chemotaxis (Fig. 5a). Consistent with a putative role in CNV, we detected robust *Il1b* mRNA expression that colocalized to *Adgre1* mRNA (encoding the Mϕ marker F4/80) in CNV lesions of *Alu* RNA-treated eyes (Fig. 5b). Intravitreous administration of an IL-1β nAb reduced Mϕ accumulation (Fig. 5c) and day three CNV volume (Fig. 5d) in *Alu* RNA-treated eyes. We sought to determine whether combined administration of nAbs targeting Vegfa and IL-1β reduced CNV volumes in an additive manner. Intravitreous administration of nAbs against either Vegfa or IL-1β reduced day seven CNV volumes (Fig S7). Combined administration of low dose Vegfa nAb (1 ng) and IL-1β nAb reduced CNV volume to a greater extent than low dose Vegfa nAb alone; interestingly, combined administration of high dose Vegfa nAb (5 ng) and IL-1β nAb had no further reductive effect compared to high dose Vegfa nAb alone (Fig. S7). These findings suggest that while the effects of Vegfa and IL-1β inhibition appear to overlap, combining these two treatments may result in some increased therapeutic effect under specific conditions.

Mϕ inflammasome activation and IL-1β production could conceivably promote angiogenesis in two non-mutually exclusive ways. First, inflammasome agonists enhance Mϕ ingression (Fig. 4), which may be sufficient to drive increased angiogenesis. In addition, an inflammasome agonist could enhance the angiogenic potential of ingressed Mϕ. To test these concepts, an ex vivo choroidal sprouting assay was used as previously described [32, 33]. Three days after seeding choroid pieces from WT mice in growth factor-reduced Matrigel, an equal number of mock- or *Alu* RNA-transfected BMDM were added to each developing sprout and sprout size was quantified on day six. Consistent with previous reports [33], adding BMDM led to enhanced choroidal sprouting (Fig S6). However, *Alu* RNA-transfected BMDM did not further exacerbate sprout growth compared to mock-transfected BMDM (Fig S6). We interpret this finding to mean that inflammasome activation does not enhance the intrinsic angiogenic potential of BMDM, but rather exacerbates angiogenesis by increasing the extent of Mϕ ingression.

## Discussion

We report a new method by which to interrogate the consequences of inflammasome activation in pathological neovascularization using experimental laser CNV. Immediately following laser thermal injury, subretinal administration of several inflammasome agonists exacerbated CNV volume in WT mice. This effect was abrogated by genetic deletion and pharmacological inhibition of the inflammasome activator P2X7, constituents of the NLRP3 inflammasome, and the adaptor MyD88. To assess the role of macrophage inflammasome in CNV, myeloid-specific caspase-1 knockouts underwent *Alu* RNA subretinal injection post-laser injury and demonstrated significantly less lesion volume and F4/80 + macrophage recruitment. We also observed inflammasome activation that colocalized to F4/80 + macrophages within the lesion site in retinal cryosections. To assess the role of inflammasome-mediated cytokine production in macrophage chemotaxis, conditioned media from *Alu* RNA-transfected WT and *caspase-1/11*^−/−^ BMDM was used as the chemoattractant in a transwell migration assay. Conditioned media from WT transfected BMDM, but not caspase-1, induced chemotaxis in WT BMDM, suggesting a soluble factor arising from inflammasome activation to be the mediator of chemotaxis.

The downstream effects of inflammasome activation in mediating angiogenesis are context-specific. Whereas the work presented here as well as previous work from our group [34] and others [35] suggest a pro-angiogenic role of inflammasome activation, NLRP3 inflammasome activation has been reported to possess anti-angiogenic properties in models of hindlimb ischemia [36, 37] and ocular herpes simplex virus 1 infection [38]. Inflammasome activation in macrophages was critical to the *Alu* RNA-exacerbated laser CNV phenotype, possibly due to the synergistic role of VEGF and IL-1β in promoting angiogenesis [31].

The findings presented here suggest an experimental approach to help bridge the disparate observations that, while inhibition of inflammasome constituents does not affect experimental laser CNV development, they significantly contribute to the development of CNV in several spontaneous mouse models. By simultaneously inducing CNV and administering inflammasome agonists, it is possible to assess how inflammasome activation directly contributes to CNV development. This model however is not without limitations, as the experimental laser CNV method is an injury-based approach to studying pathological ocular neovascularization. The extent to which findings apply to other ocular neovascular settings, such as CNV in human AMD or in pathologic myopia requires more investigation. Still these findings along with previous reports on the role of inflammasome in spontaneous CNV [5, 34] support the idea that inflammasome activation promotes pathological angiogenesis.

This study also adds pathological angiogenesis to the catalog of retinal pathologies in which inflammasome is implicated, which previously included RPE cell death, retinal degeneration, and neurovascular dysfunction in diabetes [2, 39, 40]. By implicating inflammasome directly in pathological angiogenesis, our findings support further investigation into inflammasome as a therapeutic target for pathological angiogenesis in retinal diseases such as AMD, diabetic retinopathy, and retinopathy of prematurity, as well as in other contexts of pathological angiogenesis such as cornea and solid tumors.

## Supplementary Material

Supplementary Methods and Figures

## Data Availability

Data that support the figures and conclusions presented in the manuscript will be provided upon reasonable request to the corresponding author.
