# Supplementary material for: Inflammasome activation aggravates choroidal neovascularization
Source: Angiogenesis. 2024 Sep 24;27(4):919–29. doi: 10.1007/s10456-024-09949-1 (PMC11563918; doi:10.1007/s10456-024-09949-1)
Supplement: Supplementary file 1 — Supplementary Material 1 [file 10456_2024_9949_MOESM1_ESM.pdf]

Journal: Angiogenesis

Inflammasome activation aggravates choroidal neovascularization

Ryan D. Makin[1, 2, 3], Ivana Apicella[1, 2], Roshni Dholkawala[1, 2], Shinichi Fukuda[4], Shuichiro Hirahara[5], Yoshio Hirano[5], Younghee Kim[1, 2], Ayami Nagasaka[1, 2], Yosuke Nagasaka[1, 2], Siddharth Narendran[6], Felipe Pereira[7], Akhil Varshney[1,2], Shao-bin Wang[1, 2], Jayakrishna Ambati [1, 2, 8, 9], Bradley D. Gelfand[1, 2, 10, *]

[1] Center for Advanced Vision Science, University of Virginia School of Medicine, Charlottesville, VA 22903

[2] Department of Ophthalmology, University of Virginia School of Medicine, Charlottesville, VA 22903

[3] Molecular and Cellular Basis of Disease Graduate Program, University of Virginia School of Medicine, Charlottesville, VA 22903

[4] Department of Ophthalmology, University of Tsukuba, Tsukuba, Ibaraki 305-8575, Japan

[5] Department of Ophthalmology and Visual Science, Nagoya City University Graduate School of Medical Sciences, Nagoya, Japan

[6] Aravind Eye Care System, Madurai, India

[7] Departamento de Oftalmologia e Ciências Visuais, Escola Paulista de Medicina, Universidade Federal de São Paulo, São Paulo, Brazil

[8] Department of Pathology, University of Virginia School of Medicine, Charlottesville, Virginia 22903

[9] Department of Microbiology, Immunology, and Cancer Biology, University of Virginia School of Medicine, Charlottesville, Virginia 22903

[10] Department of Biomedical Engineering, University of Virginia School of Engineering, Charlottesville, Virginia 22903

*Corresponding author: Bradley D. Gelfand, Center for Advanced Vision Science, Department of Ophthalmology, University of Virginia School of Medicine, 415 Lane Road, MR-5 Room 3131, P.O. Box 801319, Charlottesville, VA 22908; gelfand@virginia.edu

SUPPLEMENTAL METHODS

Protein isolation and western blotting

Peripheral BMDMs were obtained from WT, *Casp1*$^{loxP/loxP}$, and *Casp1*$^{loxP/loxP}$ x *LysM*-Cre mice and lysed in radio-immunoprecipitation (RIPA) buffer (Millipore Sigma, Rockville, MD, US; R0278-500ML) with protease/phosphatase inhibitors (ThermoFisher, A32963). Protein concentration was quantified with the Pierce BCA Protein Assay Kit (ThermoFisher, 23225). Protein samples were separated on a 4-20% SDS- PAGE gel (Bio-Rad; 4561094) and transferred to a 0.45 Mm Immobilon-P PVDF membrane (Millipore Sigma; IPVH00010). After incubation for 1 hour in LICOR Intercept blocking buffer (LICOR, Lincoln, Nebraska, US; 927-70010), membranes were immunoblotted with anti-Caspase-1 (p20) antibody, 1:500 (AdipoGen; clone Casper-1, AG-20B-0042-C100), anti-a-tubulin, 1:5000 (Millipore Sigma; clone DM1A, T6199-100UL), and visualized with IRDye 680LT Goat anti-Mouse IgG Secondary Antibody (LICOR; 926- 68020).

Choroid sprouting assay

The choroid sprouting assay was performed as previously described [1], with minor modifications. Briefly, 1 mm^2 peripheral choroid tissue explants from WT mouse eyes were seeded into 30 µL growth-factor reduced Matrigel (Corning Life Sciences, Corning, NY; 354230) in 6-well plates, followed by addition of Complete Classic Medium with serum (Cell Systems, Kirkland, WA; 4Z0-500). Two days after explant seeding, 20,000 mock- or *Alu* RNA-transfected WT BMDM were added to the wells in media composed of 50% Complete Classic Medium without serum (Cell Systems; 4Z3-500-S) and 50% BMDM media; control wells received the same formula media without the addition of transfected BMDM. Media was changed every day until phase contrast imaging was performed on Day 6. Explant areas were quantified in Fiji by outlining the extent of cell growth and subtracting the area of explant tissue.

REFERENCES

1. Shao Z, Friedlander M, Hurst CG, Cui Z, Pei DT, Evans LP, Juan AM, Tahir H, Duhamel F, Chen J, Sapieha P, Chemtob S, Joyal JS, Smith LEH. Choroid Sprouting Assay: An Ex Vivo Model of Microvascular Angiogenesis. PLoS ONE. 2013 Jul 26;8(7):e69552.

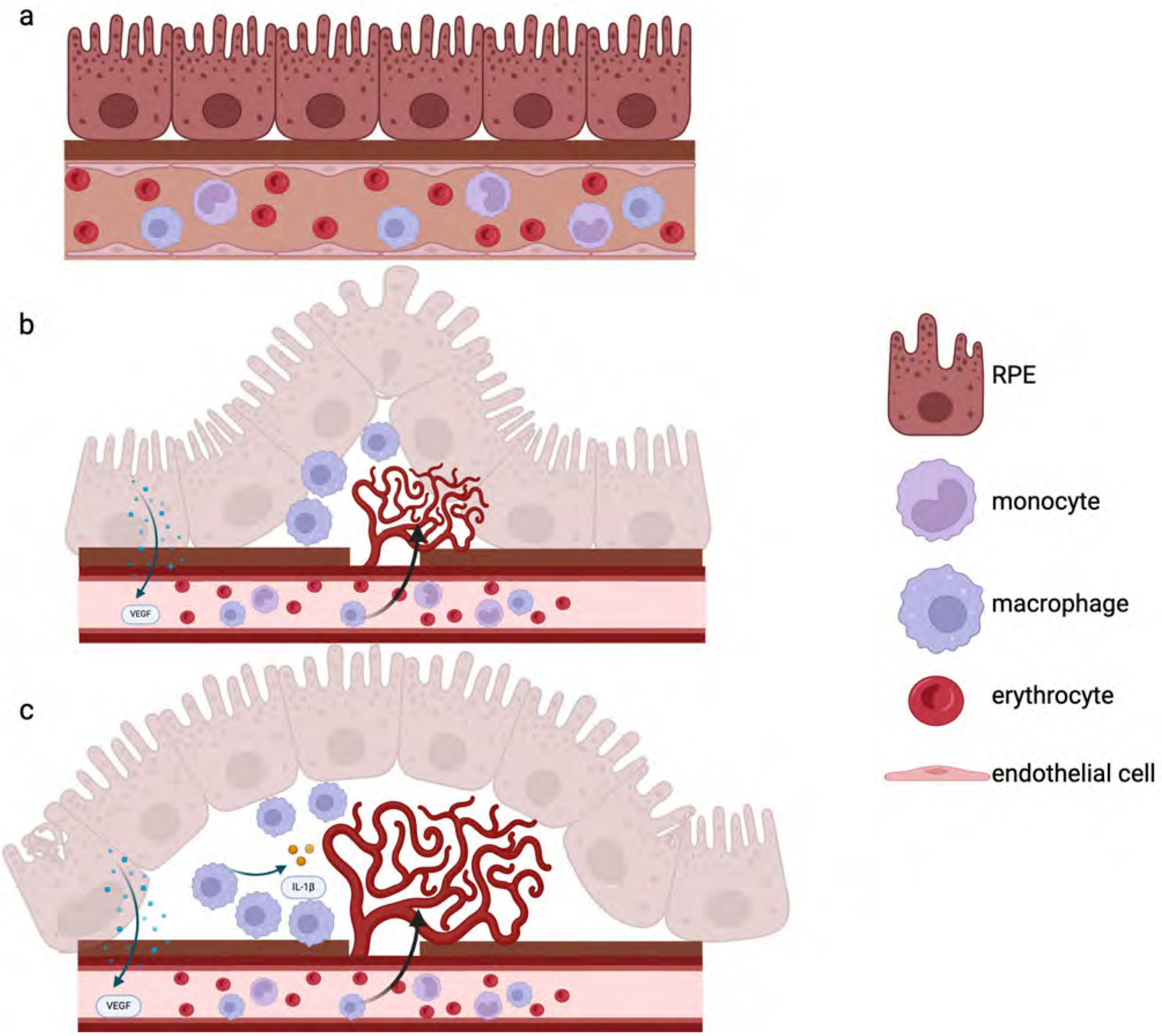

Graphical abstract. (a) Schematic of physiological outer retina. A monolayer of retinal pigment epithelial cells (RPE) provides trophic support to the overlying photoreceptors (PR). An intact Bruch's membrane (BM) provides structural support to the RPE and definition of the blood-retina barrier, with the choriocapillaris directly beneath (CC). (b) After laser injury in the absence of an inflammasome agonist, macrophages migrate to the subretinal space in response to VEGF, promoting new vessel growth. (c) In the presence of an inflammasome agonist, recruited macrophages secrete IL-1β which facilitates greater macrophage recruitment and exacerbates the neovascularization, resulting in larger CNV volumes.

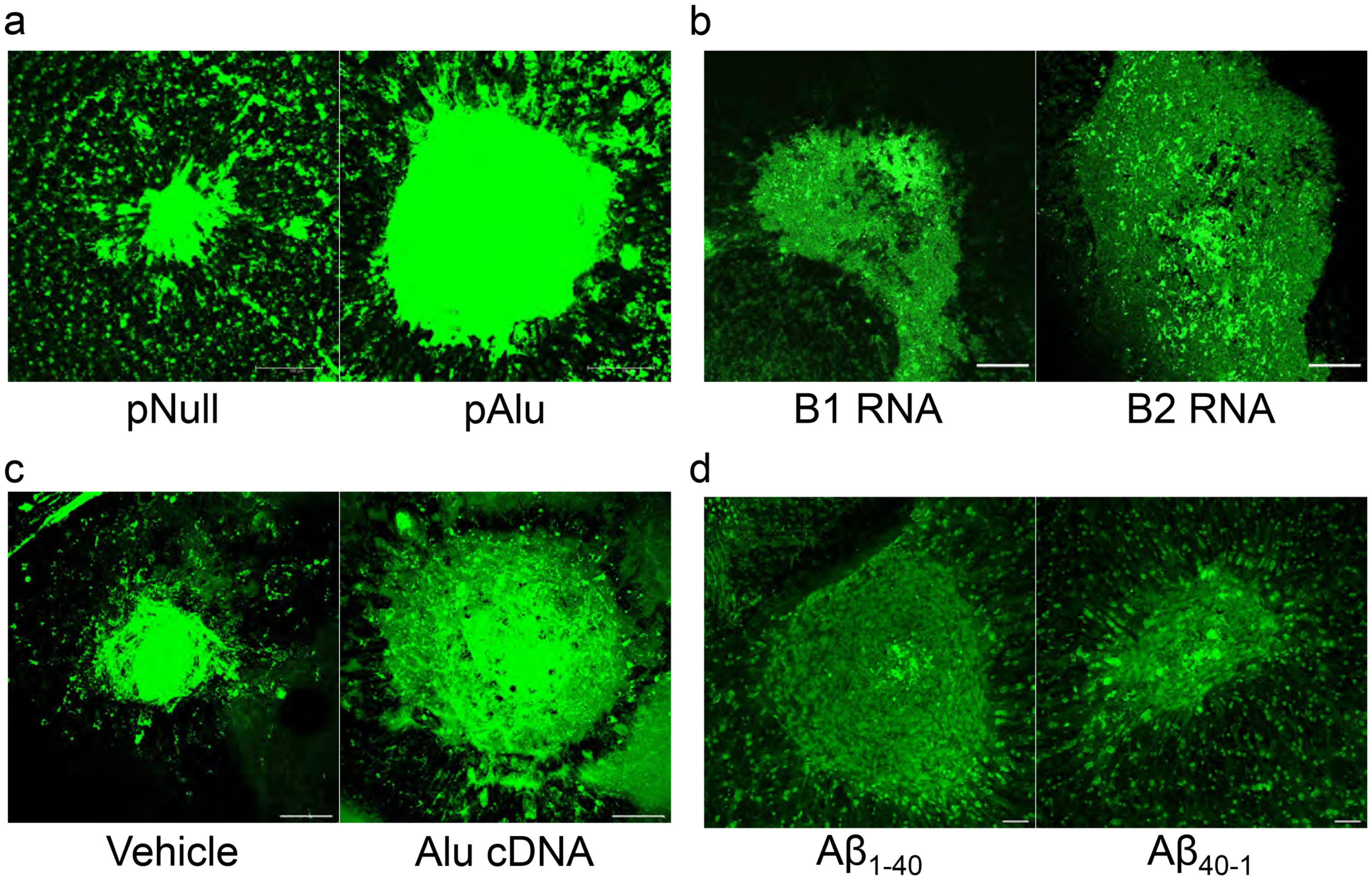

Fig. S1. (a) Representative images of IB4-labeled CNV lesions from WT mice treated with pNull or pAlu subretinal injection and laser injury corresponding to Figure 1d in the main text. (b) Representative images of B1 and B2 RNA-stimulated lesions corresponding to Figure 1e in the main text. (c) Representative images of vehicle or Alu cDNA-stimulated lesions corresponding to Figure 1f in the main text. (d) Representative images of $A\beta_{1\text{-}40}$- or $A\beta_{40\text{-}1}$-stimulated lesions corresponding to Figure 1g in the main text. Scale bars: 100 µm (a-c); 50 µm (d).

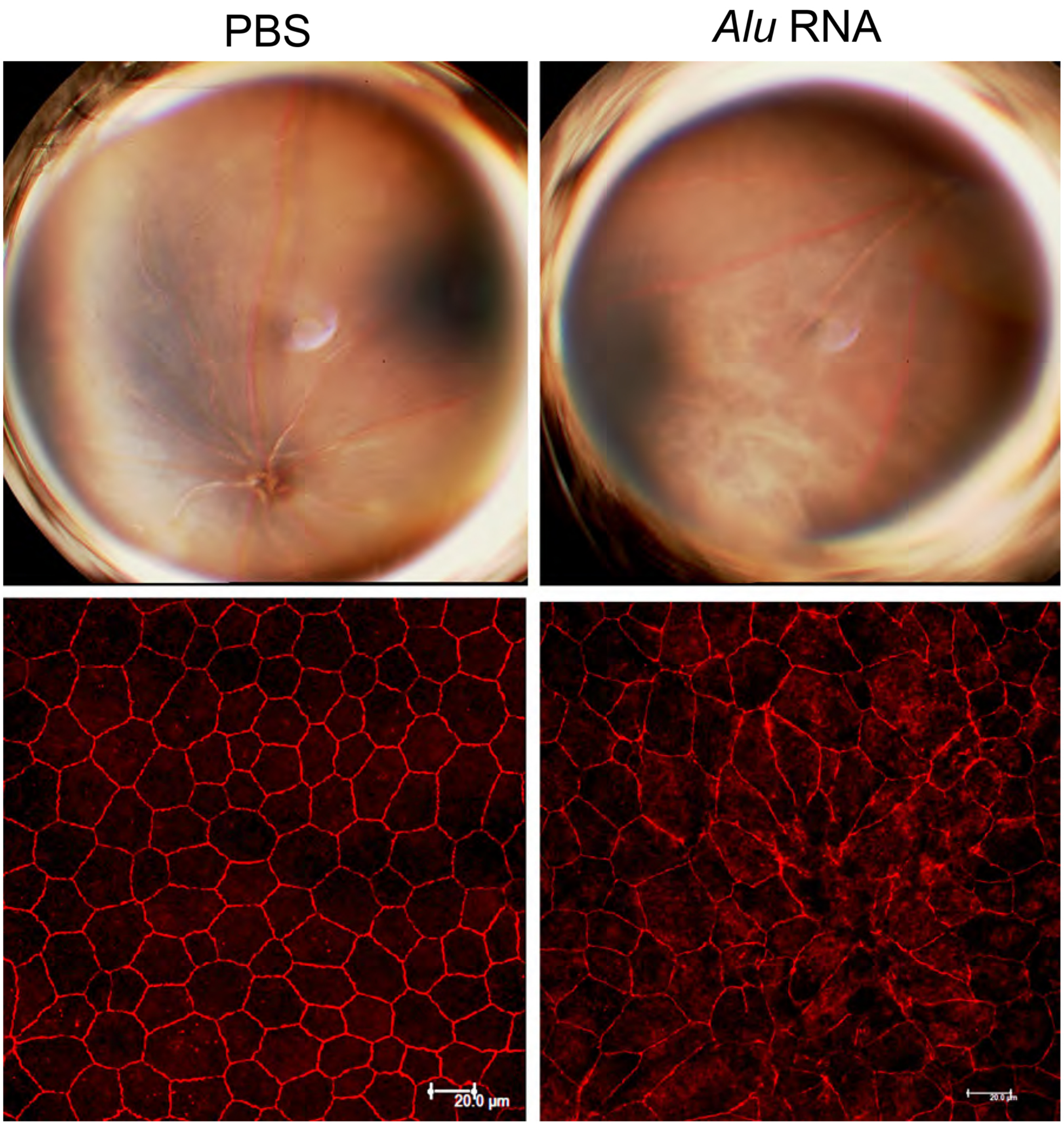

Fig. S2. Fundus images (top) and RPE flatmounts of immunolabeled ZO-1 (bottom) of *Aim2*$^{-/-}$ mice subretinally injected with PBS (left) or *Alu* RNA (right). Scale bars: 20 µm.

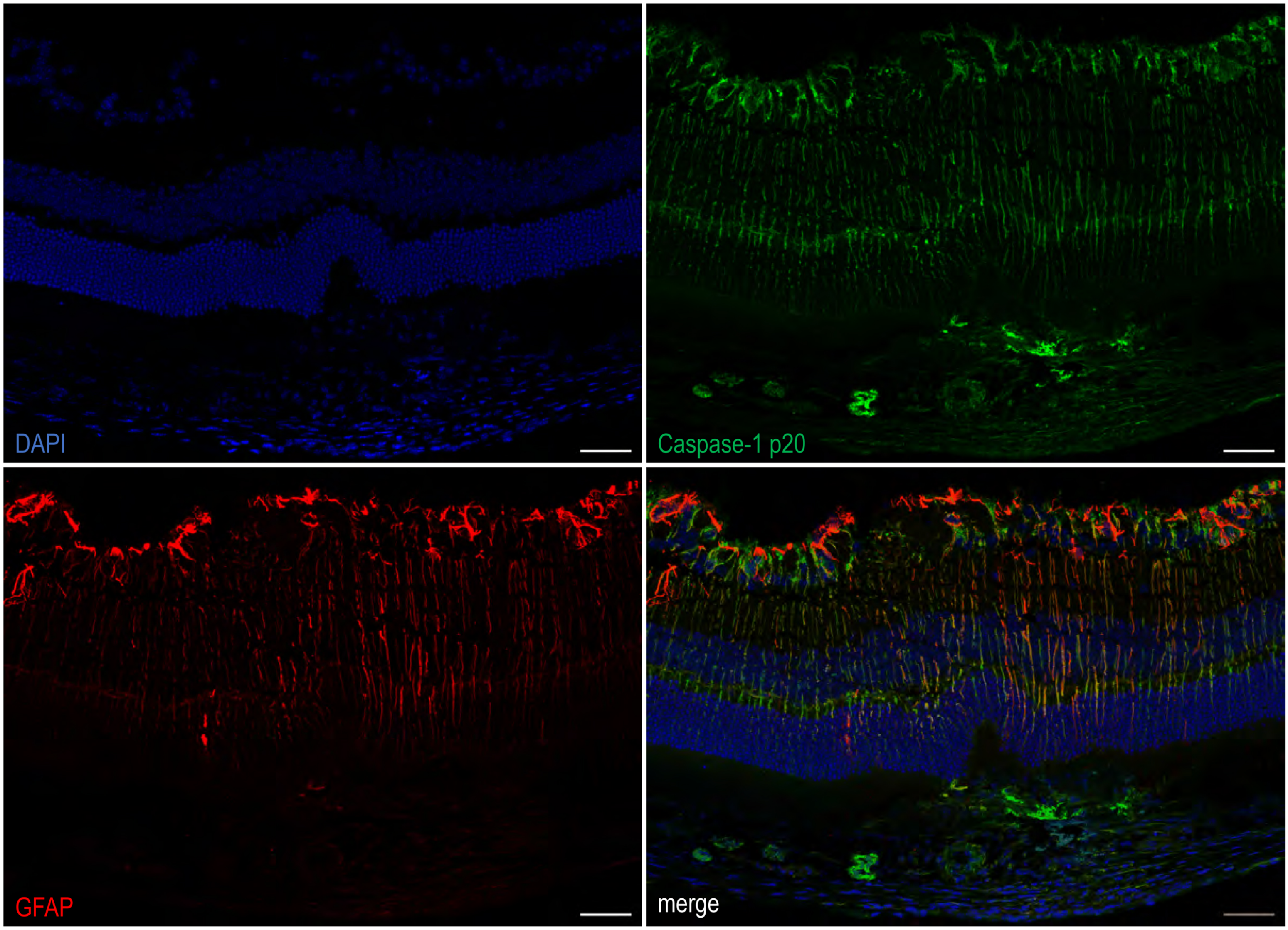

Fig. S3. Representative fluorescent micrographs of WT cryosections labeled with anti-caspase-1 p20 and anti-GFAP antibodies. Scale bar: 50 µm.

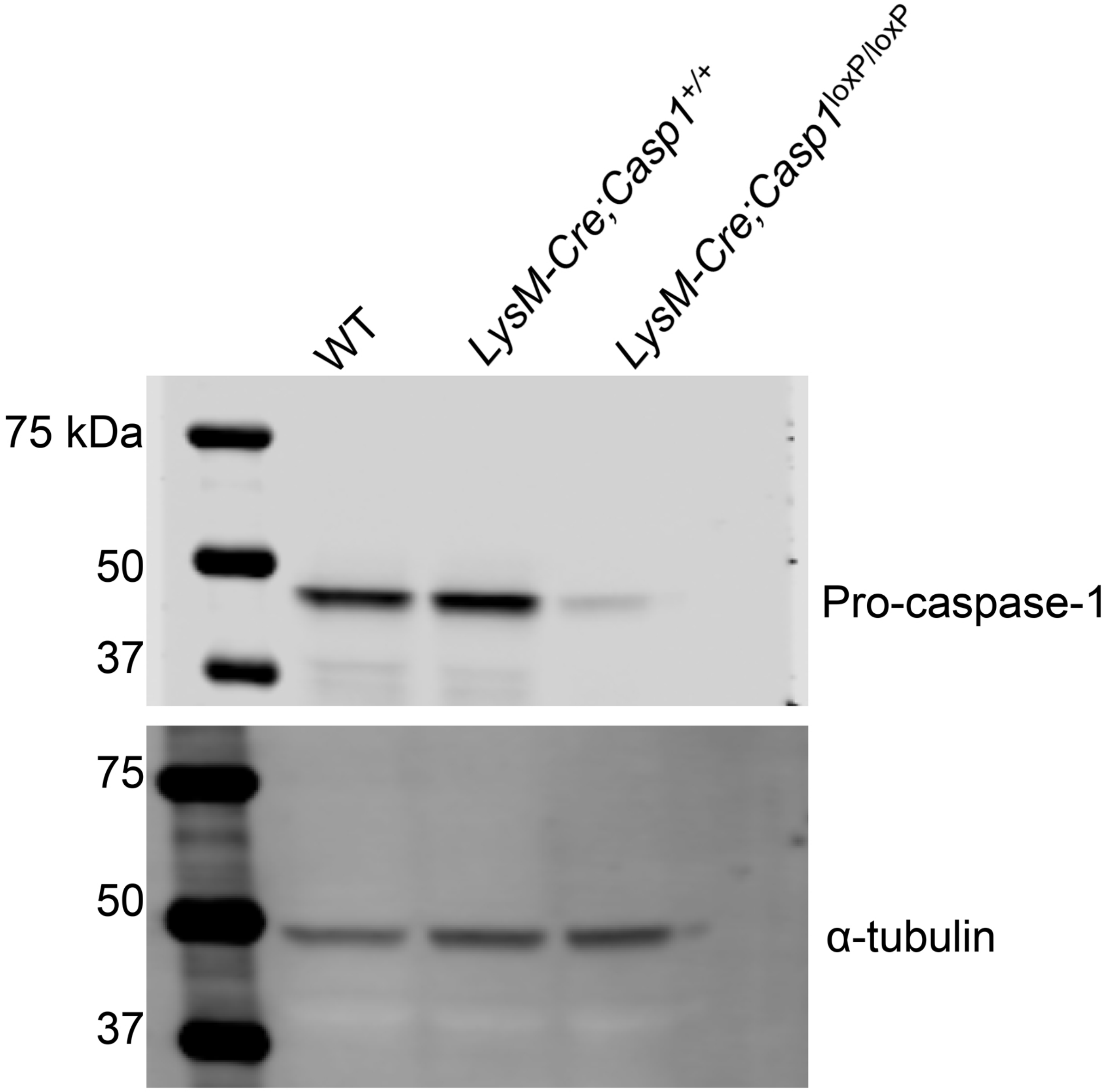

Fig S4. Representative immunoblot of pro-caspase-1 abundance in BMDM whole cell lysate isolated from WT, *LysM-Cre;Casp1*$^{+/+}$, and *LysM-Cre/Casp1*$^{loxP/loxP}$ mice. Membranes were probed with an anti-α-tubulin antibody as a loading control.

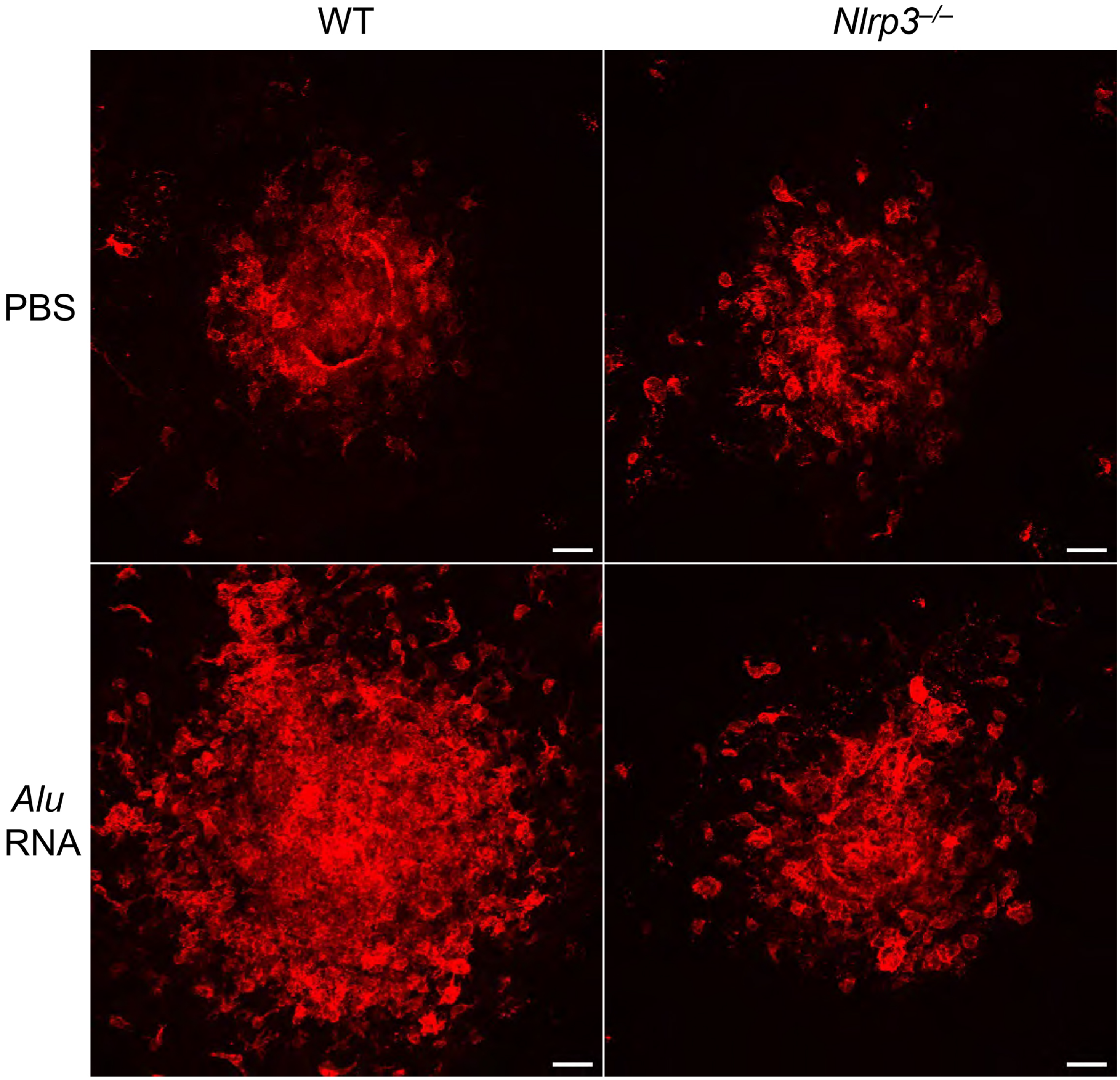

Fig. S5. Representative images of F4/80 immunolabeling on RPE/choroid whole mount**s** 3 days post-subretinal injection and laser injury, eyes from WT and *Nlrp3*$^{-/-}$ mice were enucleated and RPE/choroid complexes were immunolabeled with anti-F4/80 antibody. Scale bar: 50 µm

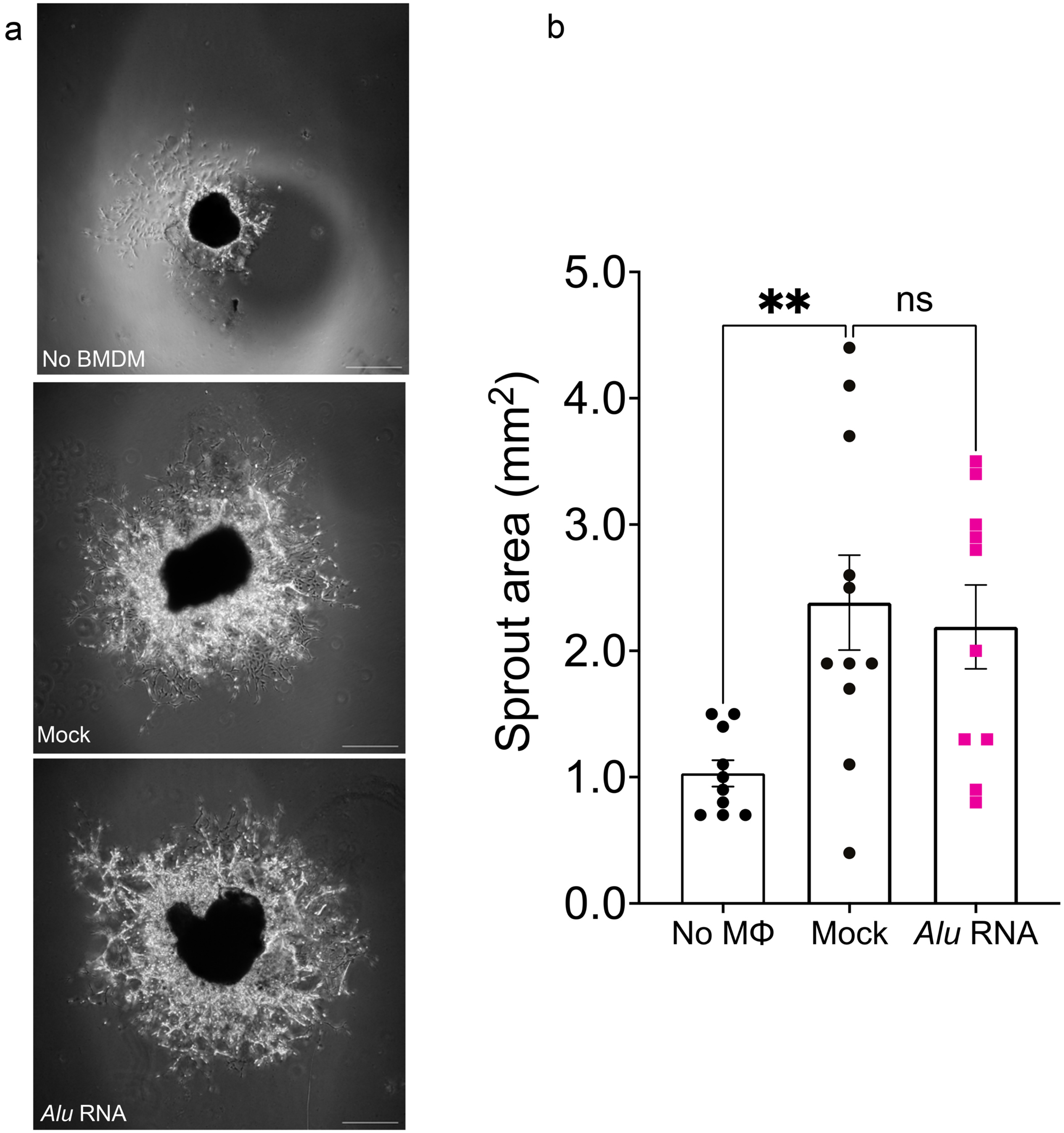

Fig S6. (a) Representative phase contrast images of choroid vessel sprouts seeded with either no BMDM (top), mock-transfected (middle) or *Alu* RNA-transfected (bottom) BMDM. (b) Quantification of choroid sprouts from (a). N=10-11. Scale bar: 500 μm.

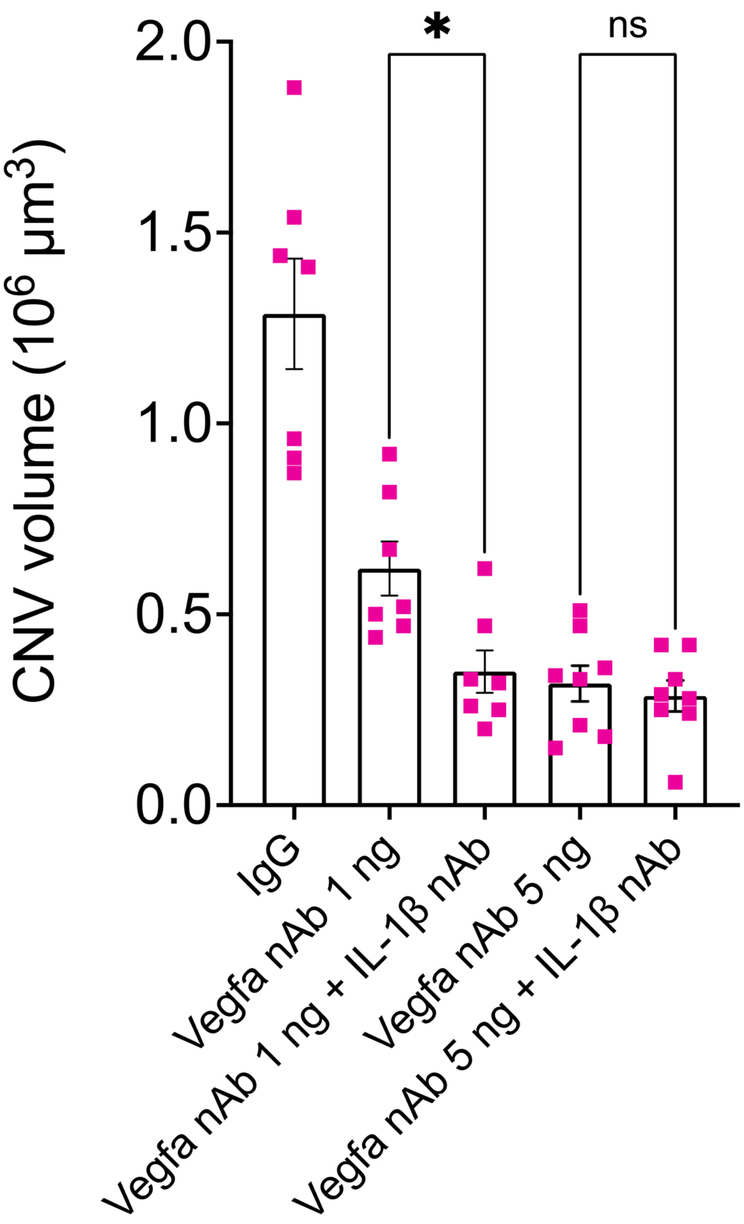

Fig S7. CNV volumes quantified 7 days post-laser injury, *Alu* RNA subretinal injection, and intravitreous injection of Vegfa neutralizing antibody with or without IL-1β neutralizing antibody.
